# Implementation of Alternative Mineral Additives in Low-Emission Sustainable Cement Composites

**DOI:** 10.3390/ma14216423

**Published:** 2021-10-26

**Authors:** Ewa Kapeluszna, Wojciech Szudek, Paweł Wolka, Adam Zieliński

**Affiliations:** 1 Faculty of Materials Science and Ceramics, AGH University of Science and Technology, 30-059 Kraków, Poland; szudek@agh.edu.pl; 2 Astra Technologia Betonu Sp. z.o.o, 83-010 Straszyn, Poland; pawel@astra-polska.com; 3 Faculty of Civil and Environmental Engineering, West Pomeranian University of Technology in Szczecin, 70-310 Szczecin, Poland; adam.zielinski@zut.edu.pl

**Keywords:** supplementary cementitious materials, mineral additives, cement hydration, zeolite, diatomite, bentonite, trass, sustainability

## Abstract

The influence of four naturally occurring mineral additives (zeolite, diatomite, trass and bentonite) on the hydration and properties of cement pastes and mortars was investigated. The materials change the phase composition, heat of hydration (determined by calorimetry) and mechanical properties of composites. After 28 days, the amount of Ca(OH)_2_ was reduced by up to 23% and up to 35% more C-S-H was formed, as proved by TG measurements. Differences were observed in the kinetics of heat release, especially for 25% of the addition. In the calorimetric curves, an additional exothermic effect is observed, related to the alteration in the hydration of C_3_A in cement. From the point of view of beneficial influence on mechanical properties of mortars, the additives could be ranked as follows: bentonite < diatomite, zeolite < trass after 2 days and bentonite < diatomite < trass < zeolite after 28 days of curing. The highest compressive strength (58.5 MPa) was observed for the sample with a 10% addition of zeolite. Zeolite, trass, bentonite and diatomite are all pozzolanic materials; however, their activity varies to an extent due to the differences in their specific surface area and the content of the amorphous phase, responsible for the pozzolanic reaction.

## 1. Introduction

Recently published research [1,2,3,4] clearly indicates a demand for new supplementary cementitious materials (SCMs) that could be potentially introduced as components of common cements, alongside well-described additives of anthropogenic origin that are currently used in the cement industry–mainly siliceous and calcareous fly ashes (FA), granulated blast furnace slags (GBFS) and finely ground limestone. The supply of the first two materials is gradually decreasing as a result of the climate-related transformation of the European energy sector and soon, it will be insufficient to cover the market demand [5], which in the following years might lead to an increase in the price of concrete or ever a downturn in the whole construction sector. The use of alternative SCMs on the industrial scale is possible on the condition that their resources are appropriate and their properties are competitive against traditional additives, which are typically by-products of various industries [6].

European standards, including EN 206, allow the use of fine-grained mineral additives in concrete in order to improve or obtain certain features of the material. Type II additives are characterized by pozzolanic or latent hydraulic properties. However, the exact standard requirements are given only for fly ashes (EN 450-1), ground granulated blast furnace slags (EN 15167-1) and silica fume (EN 13263-1). The use of alternative SCMs in concrete technology requires comprehensive research, as there are no official guidelines that would allow a straightforward assessment of their applicability. Moreover, recent research is focused also on the incorporation of mineral additives in non-standard, innovative concrete applications, such as 3D printing [7,8,9].

Pozzolans contain either only silicon or both silicon and aluminum oxides. In the hydrating cement system, if the material is of sufficient fineness, the oxides react with calcium hydroxide, forming calcium silicate hydrates (C-S-H phase). Pozzolanic activity usually depends on the content of glassy silica, which is created as a result of rapid cooling. Therefore, most natural pozzolans are of volcanic origin [10]. Some natural mineral additives exhibit high pozzolanic activity merely after grinding. Others require additional thermal treatment in order to activate them [11,12].

Among the existing guidelines for the use of natural pozzolans, the American ASTM C618 regulations should be distinguished. Class N requirements given in the standard indicate the chemical and physical characteristics that are imposed of raw or calcined natural mineral additives in order to use them in concrete (Table 1). Such materials include: diatomaceous earths, opaline cherts and shales, tuffs, volcanic ashes or pumicites and other materials that require thermal treatment in order to obtain the desired pozzolanic properties, such as certain clays.

In Central Europe, a significant increase in housing and infrastructure investments is observed. The thriving market for new apartments [13] and the rapid development of road and railway systems result in a continuously growing demand for concrete. However, the European and global trends aimed at keeping global warming below 2 °C, compared to the pre-industrial levels, dictate a reduction in carbon dioxide emissions, considered the main cause of the increasing average annual temperature [1]. Hence, changes are introduced in order to lower the production of Portland clinker and replace the conventional, coal-fired power plants with renewable energy sources. The latter results in the decreasing supply of good quality fly ashes [14], commonly used in cement and concrete manufacturing. Therefore, from the sustainable development point of view, it seems necessary to search for alternative materials that could partially substitute Portland clinker in cement without significantly deteriorating the properties of hardened composites, as it would allow people to:

- reduce the CO_2_ emissions related to cement production,
- recycle certain industrial wastes,
- improve the properties and durability of hardened concrete,
- reduce the cost of cement and concrete manufacturing.

A variety of alternative supplementary cementitious materials are available worldwide. However, their properties and suitability depend on the origin and condition in which they are used. The already tested, non-standard alternative mineral additives include i.a. ground waste expanded perlite [15,16], waste glass powder [17,18], fluidized bed combustion fly ashes [19,20] or alkali-activated waste or synthetic glasses [21,22].

Prior research indicates a high potential of zeolite as a partial substitute of cement in concrete. According to Ahmadi et al. [23], replacing up to 20% of OPC with natural zeolite allows for a 24% and 23% increase in the compressive strength of concretes after 28 and 90 days of curing, respectively. Moreover, after 28 days, the expansion of the composites was reduced by up to 95% and their permeability has significantly decreased, which resulted in a lower water absorption and chloride diffusivity, contributing to the improved durability of the material. Similar conclusions were reached by Ghafari et al. [24], who additionally used TGA to prove that after 28 days of hydration, the addition of zeolite increases the amount of water chemically bound in C-S-H by 25%. On the contrary, Markiv et al. [25] reported that substituting 10% of cement with natural zeolite (clinoptilolite) slightly decreases the mechanical performance and increases the water absorption of concretes; however, it improves their freeze-thaw resistance and reduces their drying shrinkage.

Bentonite, consisting mainly of montmorillonite, was also investigated as a possible partial substitute for Portland cement. According to Ahmad et al. [26], its pozzolanic activity index (determined according to ASTM C618) exceeded 75%, meeting the requirements of the standard. After 28 days, the compressive strength of concretes dropped by 12–60% for 20–50% of cement substitution. The addition of 30% of the material allowed to decrease the water absorption and improve the sulfate resistance of the composites. However, bentonite severely decreases the consistency of fresh concrete mix–the slump flow value of concrete in which 20% of OPC was substituted with the material was approx. 38% lower, compared to the reference sample. Similar findings were reported by Mirza et al. [27].

Trass, excavated from the volcanic tuff deposits, is another material characterized by pozzolanic properties [28]. However, few studies were dedicated to its performance as a sole substitute for Portland cement in traditional mortars and concretes. According to Joshaghani [29], after 90 days of curing, the compressive strength of mortars with 10–30% of trass in the binder was similar to the reference value (the differences were in the range of 1–4%). However, after only 28 days, the mechanical properties of all of the trass-containing mortars were inferior to the control sample. For 10–30% of OPC substitution, trass limits the expansion related to the alkaline reactivity of carbonates by 62–94% after 56 days of curing.

Diatomite, also known as diatomaceous earth, is a natural pozzolan. According to Degirmenci et al. [30], when used as a substitute for 5–15% of OPC in mortars, the material contributed to a 3–48% drop in the compressive strength after 56 days of curing. However, as a replacement for 5% of Portland cement, it allows to obtain mechanical properties similar to the reference sample while improving the sulfate resistance of mortars. Yilmaz [31] reported that after both 28 and 360 days of hydration, the compressive of mortars containing up to 10% of diatomite blended with OPC was comparable to the reference sample, while for 20% of the substitution, a 24% decrease was observed. The water demand of the binder increased from 28.4 wt.% to 30.0–37.0 wt.% for 10–20% of the addition, while the initial setting time was shortened by 10–15 min. On the contrary, Ahmadi et at. [32] determined that up to 40% of Portland cement can be substituted with raw diatomite without any deterioration in compressive strength after 28 days of curing. Moreover, after 91 days, the modified samples were up to 13% stronger, compared to the control mortar. The water absorption of the composites has decreased by 1–22%, which should contribute to their improved durability. However, the addition had a negative effect on the consistency of fresh mortars–their slump values were 5–14% lower, compared to the reference sample.

Studies have shown that, taking into account the water demand and setting time of cements, mineral additives characterized by a grain size distribution similar to CEM I 42.5R cement and high pozzolanic activity are the most favorable. Even though there are standards such as ASTM C311 and C618 that allow to roughly evaluate the potential pozzolanic activity of a material, no chemical method truly reflects the proportion of active oxides in SCMs. Therefore, the comprehensive experimental results presented in this article, including the chemically bound water and calcium hydroxide content and the difference between early and late relative strength, allow to compare and select the most favorable components for sustainable cement composites.

The goal of the research was to compare the properties of the selected alternative mineral additives of natural origin and to evaluate their applicability as main components of common cements (SCMs). The use of such materials allows to obtain more sustainable, low-emission cements due to their reduced content of Portland clinker, the production of which is the most emissive. It is important for the manufacturers that these additives have no negative impact on the standard properties of the composites. Sometimes, however, they can improve certain features (due to their pozzolanic activity).

The article presents the results of research on the influence of alternative mineral additives on the properties of cements and mortars. Chemical and phase compositions of the SCMs were established to estimate the content of potentially reactive compounds. Initial setting time, water demand and heat evolution of modified cements were evaluated. Consistency and mechanical properties of fresh and hardened mortars and the C-S-H/portlandite contents in hydrated pastes were determined. A detailed comparison of the materials in one experiment allows to choose the most suitable additive for future applications and shows the directions of changes in the parameters of building materials. The results are important from a practical point of view as they facilitate the application of new and not fully characterized materials.

## 2. Materials and Methods

### 2.1. Methods

Chemical composition of raw materials was determined using a WDXRF Axios mAX spectrophotometer equipped with a 4 kW Rh anode X-ray tube. XRD patterns were collected with a PANalytical Empyrean diffractometer equipped with a Cu/Ni lamp operating at 35 kV/16 mA. Step size was 0.05°2θ. Phase composition was analyzed using X’Pert Highscore Plus software. Particle size distribution was studied with a Malvern Mastersizer 2000 laser diffractometer using ethyl alcohol as a dispersant. The specific surface area of the additives was determined by BET method (gas adsorption isotherm analysis). The degassing temperature was 120 °C.

Mix proportions of pastes subjected to thermal analysis and hydration kinetics evaluation are presented in Table 2. The pastes were prepared by mixing standard CEM I 42.5R Portland cement with each of the studied mineral additives in the amount of 10% and 25%. Water to binder ratio was 0.5. The samples were casted in polyethylene containers, sealed to prevent water loss and carbonation and cured at room temperature (20 ± 3 °C) for 28 days. In order to avoid errors in the interpretation related to cement dilution effect, two additional reference samples–Cref10 and Cref25–were prepared, containing the addition of a high purity, chemically inert ground quartz sand instead of a pozzolan. The Blaine’s specific surface area of the sand was 3500 cm^2^/g (a value typical for class 42.5 common Portland cements). Such approach allows to assume that the excess heat released during the hydration of the binder is related to the reaction of mineral additives. Heat of hydration was studied using a differential heat-conduction microcalorimeter. Thermogravimetric analysis of the hydrated samples was carried out in the temperature range of 25–1000 °C. Heating rate was 5 °C/min. No organic reagents were used during sample preparation in order to avoid the occurrence of additional thermal effects. The hydration of pastes was stopped through vacuum drying using anhydrite III as a desiccant. Samples were dried to constant mass i.e., to a daily mass change lower than 0.1%. Afterwards, they were ground in an agate mortar to a grain size below 0.63 µm and subjected to XRD and TG analyses.

Water demand and setting time of cements were determined in accordance with the EN 196-3 standard. Mortar density was measured according to EN-1015-6 and their consistency according to EN 1015-7. In order to evaluate the workability of mortars with different mineral additives, their consistency was studied 5 and 40 min after the binders were mixed with water.

Mortar samples were prepared by substituting 10% and 25% of CEM I 42.5R cement with each of the mineral additives. Mix proportions of mortars are presented in Table 3. Mortars were prepared with the desire to obtain the smallest possible differences in consistency. Their standard slump flow was in the range of 140–180 mm. Appropriate flow values of mortars containing additives characterized by increased water demand were achieved with the use of a polycarboxylate-based superplasticizer. Samples, cast in the form of bars with the dimensions of 40 × 40 × 160 mm, were cured at room temperature (20 ± 3 °C) for 28 days. Flexural and compressive strength was measured according to EN 196-1.

### 2.2. Materials Characteristics

The Portland cement used in the study was CEM I 42.5R, meeting the requirements of the EN 197-1 standard. Its most important properties are presented in Table 4.

**Figure 1:**
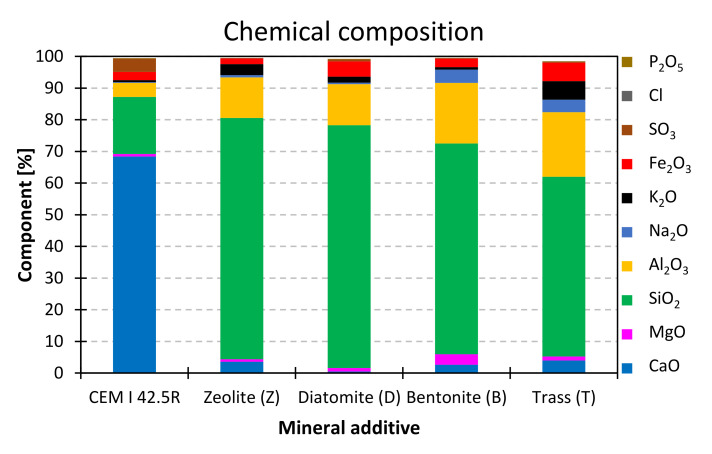
Chemical composition of cement and mineral additives.

**Figure 2:**
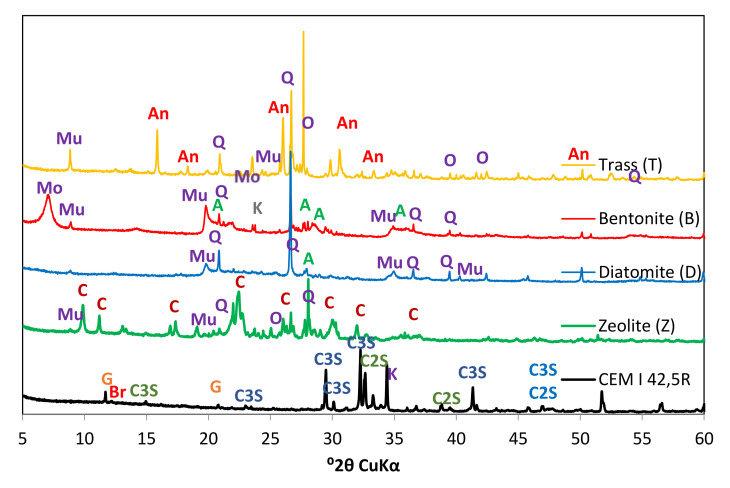
Diffractograms of studied materials: Q—quartz; An—analcime; O—potassium feldspar/orthoclase; Mo—montmorillonite; Mu—muscovite; A—albite; C—clinoptilolite; C3S—alite; C2S—belite; Br—brownmillerite; G—gypsum.

**Figure 3:**
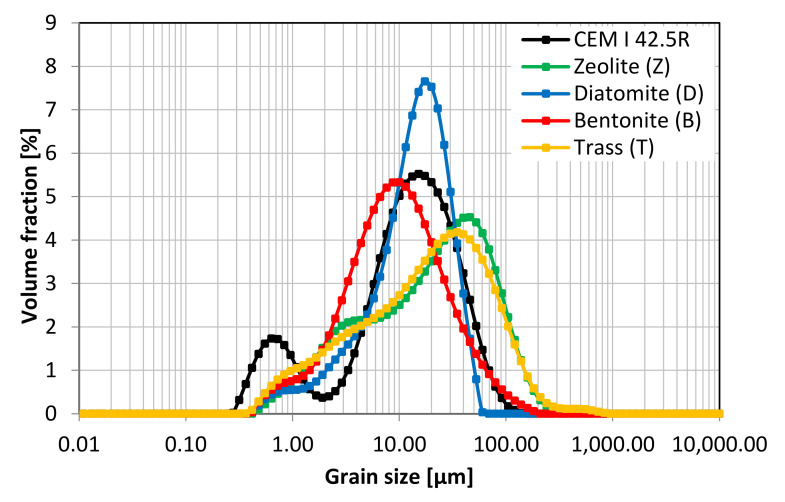
Grain size distribution of studied materials.

**Figure 4:**
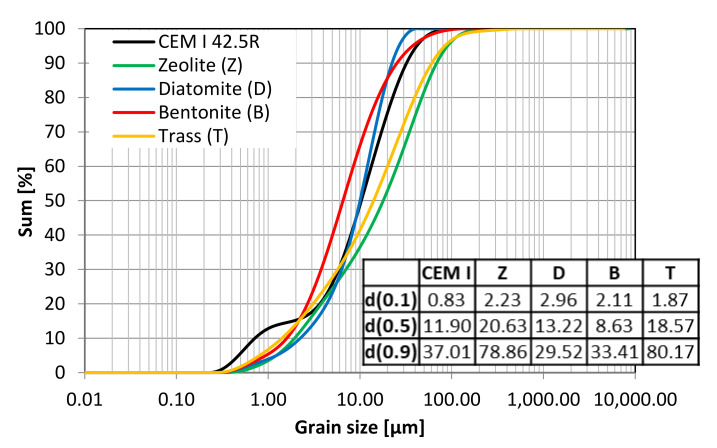
Cumulative grain size distribution curve with characteristic parameters.

Four natural mineral additives, all commercially available on Polish market, were chosen for the study. The chemical composition of cement and SCMs is presented in Table 5 and Figure 1. Table 6 presents the physical properties of the pozzolans. Phase composition of the materials was studied with the use of X-ray diffractometry. The obtained XRD patterns are presented in Figure 2. Grain size distribution of the materials is presented in Figure 3 and Figure 4.

Taking into account its standard properties, as well as the chemical and phase composition, it was concluded that the cement used in the study meets all the requirements of EN 197-1. It is a typical Portland cement, consisting of four main clinker phases: alite, belite, tricalcium aluminate and brownmillerite, which is evidenced by the presence of their characteristic reflections observed in the diffractograms (Figure 2).

All mineral additives used in the study are materials of natural origin. Chemically, they are composed mainly of silicon, aluminum, calcium and iron oxides. The alkali content in the samples is particularly important due to the risk of alkali-aggregate reaction, the products of which are expansive and can lead to cracking. If the alkali are contained in the glassy phase, they are not easily leached into the hydrating cement system and therefore, they can be considered harmless.

XRD phase analysis showed that the zeolite used in the study consists mostly of clinoptilolite–the most common zeolite on Earth. Prior research reports its positive impact on cement hydration and the compressive strength and durability of mortars and concretes [25]. Zeolite is characterized by pozzolanic activity [23], which translates into the ability to improve the late mechanical strength and reduce the porosity of the composites. The pozzolanic properties of zeolite are the result of its formation from hydrothermal deposits and through the transformation of volcanic rocks and tuffs containing active SiO_2_ and Al_2_O_3_ oxides. Small amounts of montmorillonite were also identified in the studied zeolite.

Diatomite is a siliceous sedimentary rock. In addition to the active amorphous phase present in the material, crystalline phases such as quartz and muscovite were identified in its XRD pattern. Diatomite is used in cement composites to minimize their shrinkage [30].

Bentonite is formed through the transformation of volcanic glasses and tuffs that occur in seawater. It consists mainly of montmorillonite, although it may also contain some amorphous phase characterized by pozzolanic activity. Its absorptive properties can contribute to shrinkage reduction [27].

Trass is a mineral of volcanic origin that also contains active silicon and aluminum oxides which can enter pozzolanic reaction in the environment of hydrating cement. In addition to the amorphous phase, small amounts of analcime, quartz and muscovite were identified during XRD analysis. The presence of the aluminosilicate amorphous phase, related to the geological origin of the material, is evidenced i.e., by the background elevation in the 15–35°2θ angular range of the diffractograms. Determining the amount of the amorphous phase in such materials is very difficult–no chemical method truly reflects the proportion of active oxides in SCMs.

Apart from the chemical and physical composition, the activity of mineral additives is determined by their specific surface area and grain size distribution. The results of particle size analysis, presented in Figure 3 and Figure 4, show the differences between the selected materials. Bentonite and diatomite are characterized by a tendency to agglomerate. The grain size distribution curve obtained with a laser granulometer reflects the content of both individual grains and agglomerates. Since in the production of mortars and concretes the materials are used as purchased and the standard mixing does not disperse the agglomerates, the samples were not sonicated during the measurement. Portland cement has the largest share of grains in the range of 0.2–2 µm. On the other hand, zeolite and trass are characterized by the highest content of particles in the range of 10–300 µm. The BET specific surface area of the materials is presented in Table 6. The difference in the specific surface of the materials has an impact on their homogenization, void-filling ability (filler effect [33]), chemical activity and the adsorption of water on their surface.

## 3. Results and Discussion

### 3.1. Water Demand and Setting Time

**Figure 5:**
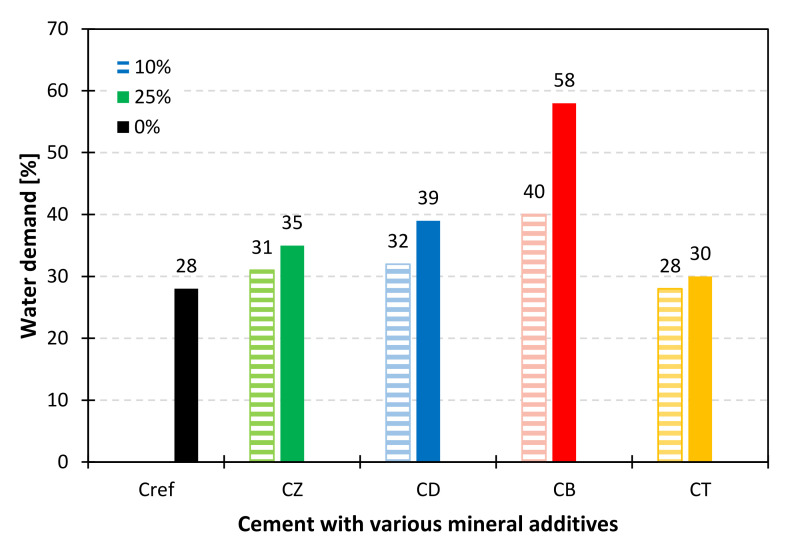
Water demand of cements with a 10% (patterned bars) and 25% (solid bars) addition of alternative SCMs.

In order to evaluate the impact of selected mineral additives on the performance of cements, water demand and setting time measurements were carried out on samples in which 10% and 25% of CEM I 42.5R OPC was substituted with zeolite, diatomite, bentonite and trass. The results (Figure 5) indicate that the most water-demanding additive is bentonite, which is related to its highest specific surface area (38.65 m^2^/g, according to BET). Results correspond to those reported by Ahmad et al. [26]. The introduction of 10% of the material increased the water demand of the binder by 43%. Therefore, it can be concluded that the use of large amounts of bentonite in mortars is unfavorable. Zeolite increased the water demand proportionally to the percentage of cement substitution. In the case of trass, a 7% increase was observed for the CT25 sample.

**Figure 6:**
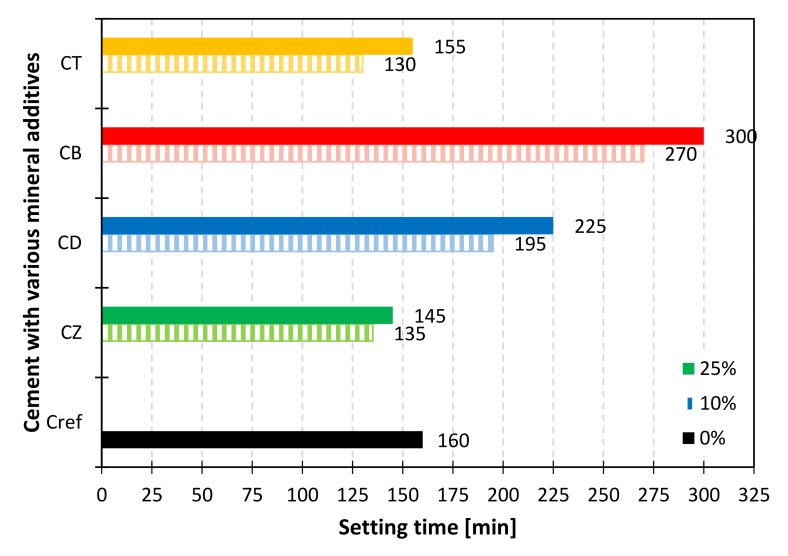
Initial setting time of cements with a 10% (patterned bars) and 25% (solid bars) addition of alternative SCMs.

Setting time depends on the degree of hydration and the specific surface area of the binder, the amount of water introduced into the system and the grain packing within the paste. Figure 6 shows the differences in the initial setting time of the reference and modified samples. As expected, the longest setting time was recorded in the case of bentonite, characterized by the lowest content of active oxides. The introduction of zeolite and trass reduced the setting time by 5–25 min, depending on the percentage of cement substitution. The effect is most likely related to their pozzolanic activity and to the water/cement ratio similar to the reference paste. Diatomite increased the initial setting time by 35 and 65 min for 10 and 25% of cement substitution, respectively.

### 3.2. Consistency

**Figure 7:**
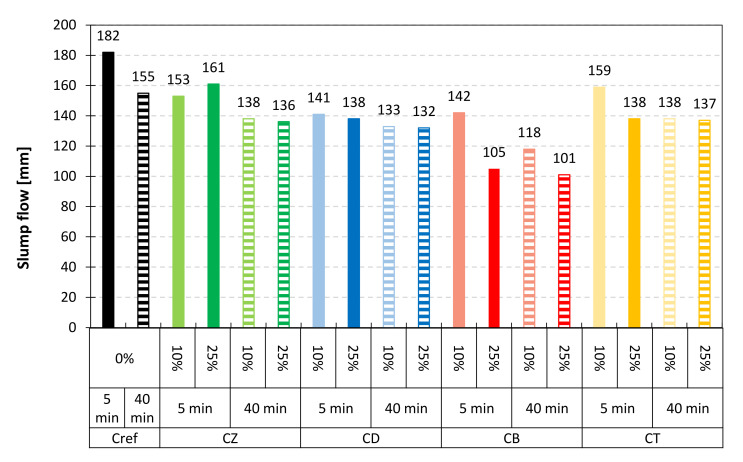
Consistency of mortars containing 10% and 25% of alternative mineral additives after 5 min (patterned bars) and 40 min (solid bars) of hydration.

**Figure 8:**
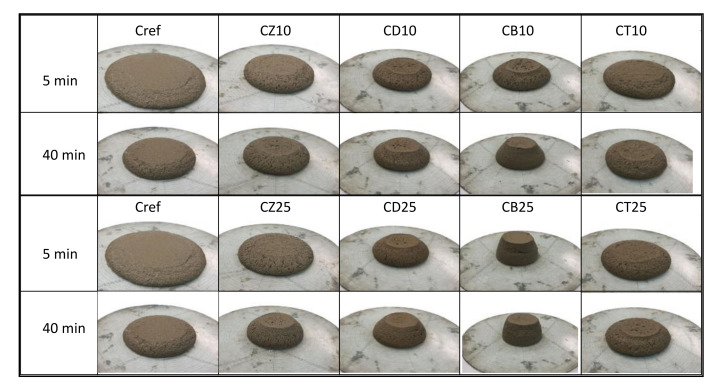
Slump flow of mortars containing 10% and 25% of alternative mineral additives after 5 min and 40 min of hydration.

The workability of mortars and concretes is related to their consistency, which affects, among other things, the pumpability of the concrete mixture and the so-called working time of mortars. The consistency of mortars was studied after approx. 5 min (according to EN 1015-7) and subsequently after 40 min in order to evaluate the variations in the rheological parameters of mixtures containing different mineral additives. Changes in consistency indicate the progress of hydration as well as the chemical activity of the materials used. The reference mortar was characterized by the highest flow diameter of 182 mm (Figure 7). The introduction of alternative mineral additives resulted in a decrease in flow, which means that the additives are more water-demanding than cement. Zeolite addition decreased the standard mortar flow by approx. 12–16%, diatomite–by 22–24% and trass–by 13–24%. The consistency of mortars containing bentonite was the driest (Figure 8)–the addition of 25% resulted in a lack of flow, while 10% decreased it by 22%. It should be noted that when bentonite was used, superplasticizer was introduced into the mortar in excess of the amount recommended by the manufacturer. Therefore, the downside of using bentonite as an additive is the entailed cost of large amounts of expensive chemical admixtures. According to the literature, overdosing superplasticizer increases the amount of air entrained in the mortar, which can have an adverse effect on strength.

### 3.3. Heat of Hydration

**Figure 9:**
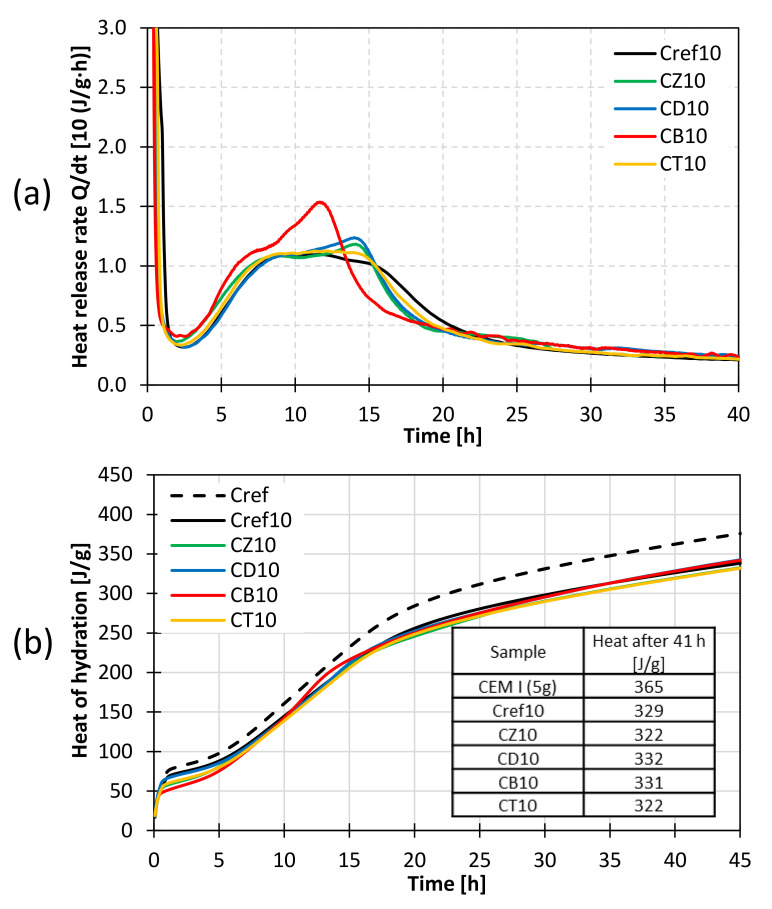
Heat of hydration of pastes containing 10% of alternative mineral additives; (**a**) heat evolution rate; (**b**) cumulative heat released as a function of hydration time.

**Figure 10:**
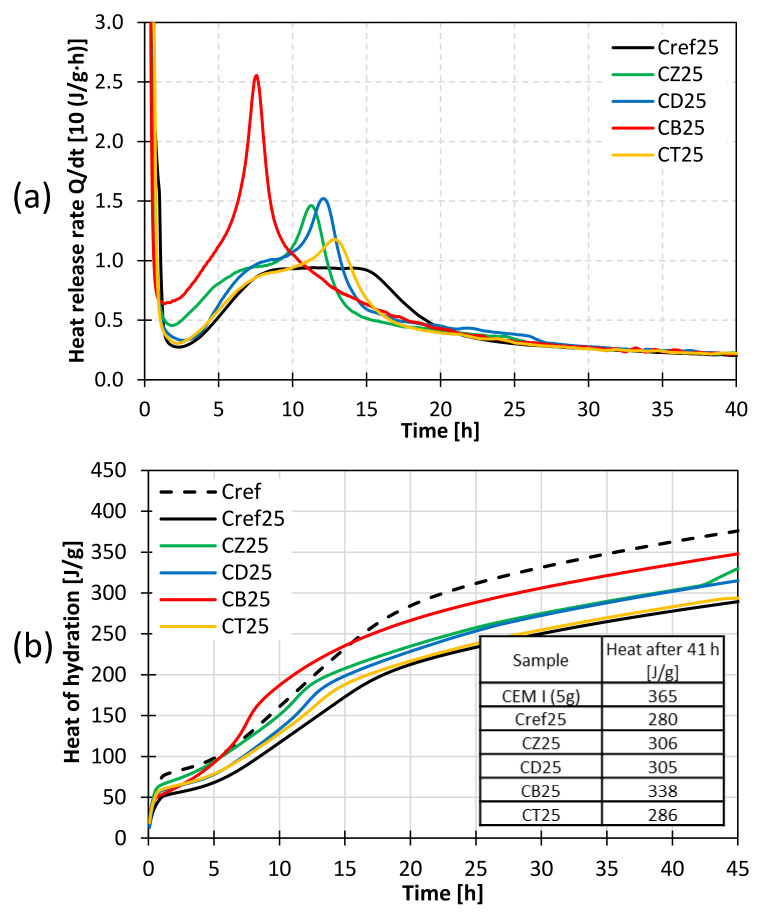
Heat of hydration of pastes containing 25% of alternative mineral additives; (**a**) heat evolution rate; (**b**) cumulative heat released as a function of hydration time.

The evolution of heat is related to the activity of the binder present in the paste and indicates the progress of the reactions taking place during cement hydration. Differential calorimetry allows for a continuous monitoring of the process. The impact of selected mineral additives on the rate of heat evolution and total heat released was investigated. In each case, 5.0 g of sample was mixed with 2.5 g of water (water/cement ratio of 0.5). Pure CEM I 42.5R OPC was used as the main reference sample (Cref). Furthermore, to eliminate the cement dilution effect, additional reference samples were prepared, in which 10% and 25% of cement was substituted with ground quartz sand as an inert component. As a result, the Cref10 and Cref25 samples contained the same amount of OPC as the binders with alternative pozzolans. The heat of hydration of pastes containing 10% and 25% of the additives is presented in Figure 9 and Figure 10, respectively.

The investigations of heat release rate showed that the mineral additives affect the hydration kinetics of cement. The amount of heat evolved by the pastes containing 4.5 and 3.75 g of cement was proportionally lower, compared to the main reference sample. The cumulative heat obtained for pastes modified with 10% of mineral additives was similar to the reference sample containing ground quartz sand (Cref10). Differences were observed in the kinetics of heat release. In the calorimetric curves (Figure 9a), an additional exothermic effect is observed between 10 and 17 h of hydration. This effect indicates an alteration in the hydration of tricalcium aluminate in cement. According to the literature, it may be related to the availability of reaction water due to the high water demand of the additives (resulting from their large specific surface area) or to the presence of aluminum in their chemical composition, which may alter the aluminate concentration in the hydrating system [34]. The effect was particularly pronounced in the case of bentonite, which is characterized by the highest specific surface area. Zeolite and diatomite also have high SSA resulting from the presence of zeolitic channels (analcime in diatomite and clinoptilolite in zeolite) and high porosity, which may account for the adsorption of water on their surface.

The introduction of 25% of mineral additives intensifies the effects observed in the calorimetric curves. The cumulative heat released by the modified cements is higher than that of the reference sample (Cref25). This may indicate the active participation of SCMs in the hydration process. Their influence can be of chemical nature, related to the early pozzolanic reaction, or it can be associated with the nucleation of the C-S-H phase (so-called filler effect), which results in a higher hydration degree of the entire system [33].

According to the most recent theories on cement hydration, it can be concluded that the presence of highly active pozzolans, characterized by large specific surface areas, affects the dissolution rate of the main clinker phase–alite, as the shape of the calorimetric curve depends mainly on its hydration. Moreover, an increase in the intensity of heat release associated with the so-called silicate effect may indirectly prove the influence of supplementary cementitious materials on the amount and structure of the precipitated hydration products. Zeolite, trass, bentonite and diatomite are all pozzolans; however, their activity varies to an extent due to the differences in their specific surface area and the content of the amorphous phase, responsible for the pozzolanic reaction.

### 3.4. Thermal Analysis

**Figure 11:**
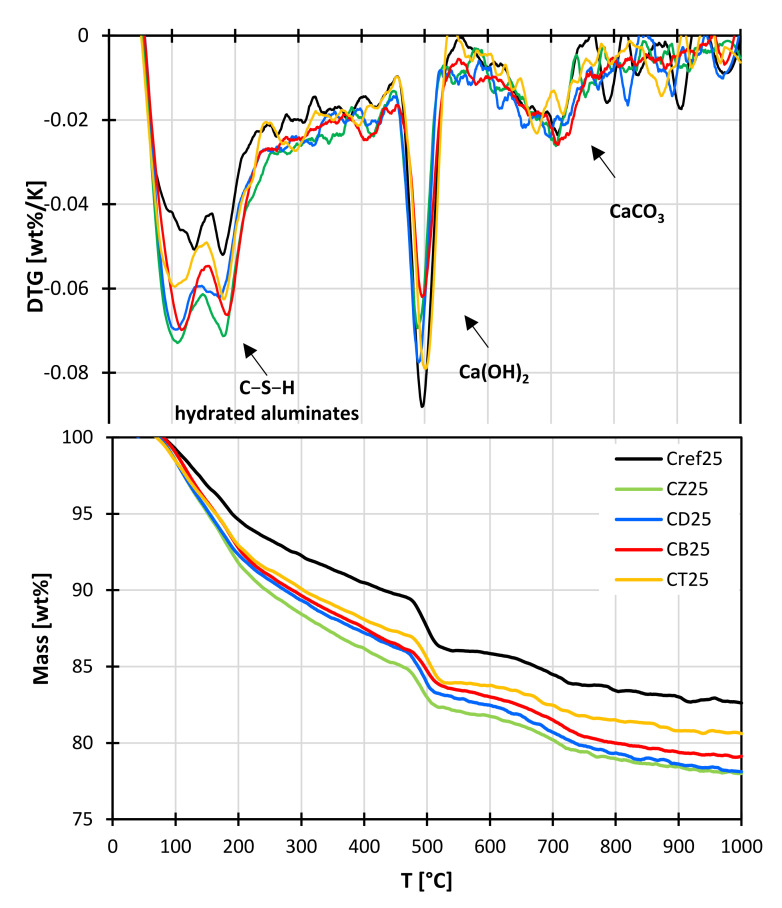
TG/DTG curves of pastes containing a 25% addition of alternative SCMs after 28 days of hydration.

The progress of the pozzolanic reaction is indicated by the decreasing calcium hydroxide content over time. Differential thermal analysis allows to compare the amount of Ca(OH)_2_ in the samples. Thermogravimetric measurements were carried out on pastes with a 25% addition of the alternative SCMs after 28 days of curing (Figure 11). The endothermic effect observed in the DTG curve in the temperature range of 450–550 °C, with a maximum at approx. 500 °C, is attributed to the weight loss associated with Ca(OH)_2_ dehydroxylation. In hydrating pastes, calcium hydroxide occurs as crystalline portlandite; however, it can also have an amorphous form. The amount of Ca(OH)_2_ in the pastes modified with mineral additives was up to 23% lower, compared to the reference sample. In the case of bentonite, the calculated hydroxide content may not reflect the true value, as the bulk density of the additive is much lower, compared to Portland cement; therefore, the same material mass occupies a much larger volume. Furthermore, during thermal decomposition of bentonite, water present between the layers of clay minerals is released, which can lead to interpretation errors. In the case of pastes containing zeolite, diatomite and trass, the endothermic effects are noticeably smaller, indicating a pozzolanic reaction of these additives. The reaction produces additional amounts of C-S-H and C-A-S-H phases, which was also confirmed by thermal analysis. The endothermic effect in the 50–400 °C temperature range is related to the loss of water chemically bound in calcium aluminosilicate hydrates and calcium sulfoaluminate hydrates (e.g., ettringite and monosulfate). The content of chemically bound water is up to 35% higher in pastes modified with mineral additives, compared to the reference sample. The highest value was obtained for the zeolite-containing paste, which indicates that it is the most active among the studied additives. Trass is the least active, probably due to the lowest specific surface area.

### 3.5. Mechanical Properties

**Figure 12:**
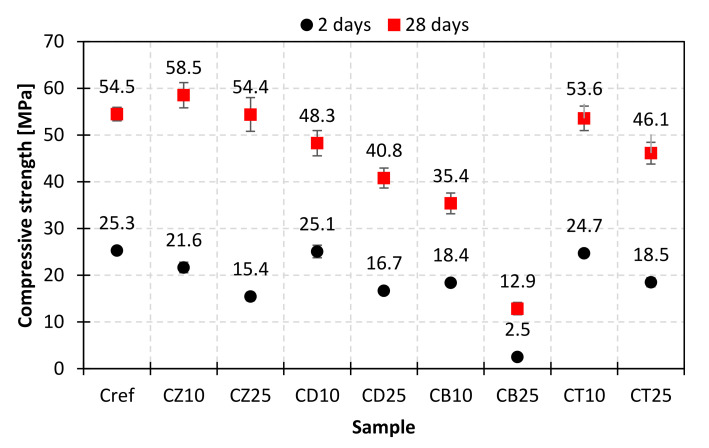
Compressive strength of mortars with a 10% and 25% addition of alternative SCMs after 2 and 28 days of hydration.

**Figure 13:**
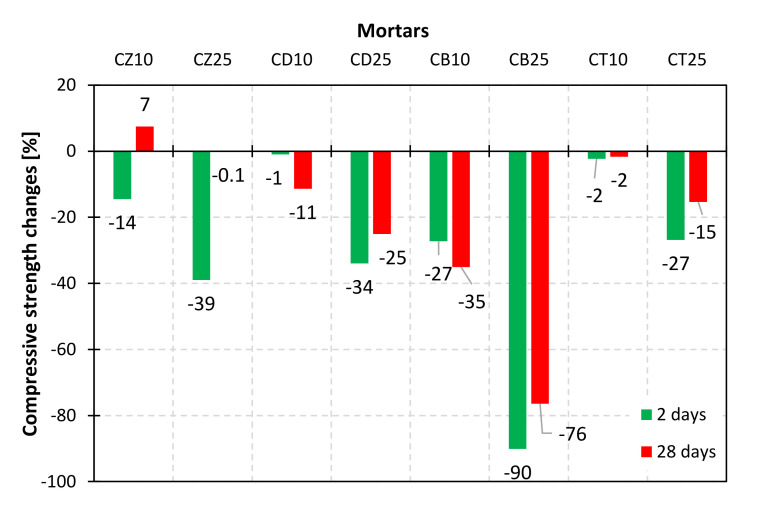
Relative changes in compressive strength after 2 and 28 days of hydration, in comparison to the reference mortar (Cref).

**Figure 14:**
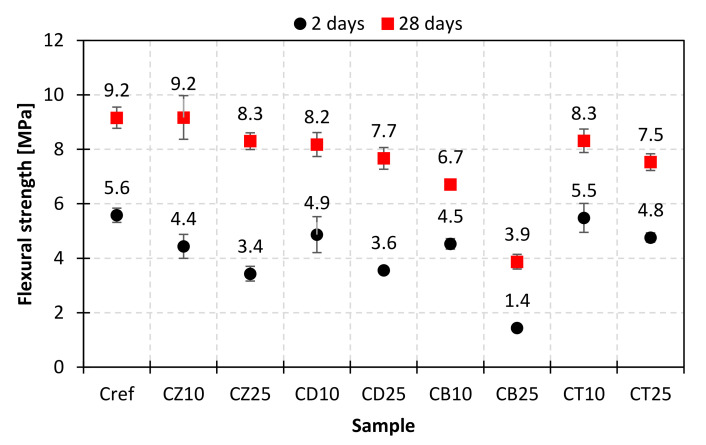
Flexural strength of mortars with a 10% and 25% addition of alternative SCMs after 2 and 28 days of hydration.

**Figure 15:**
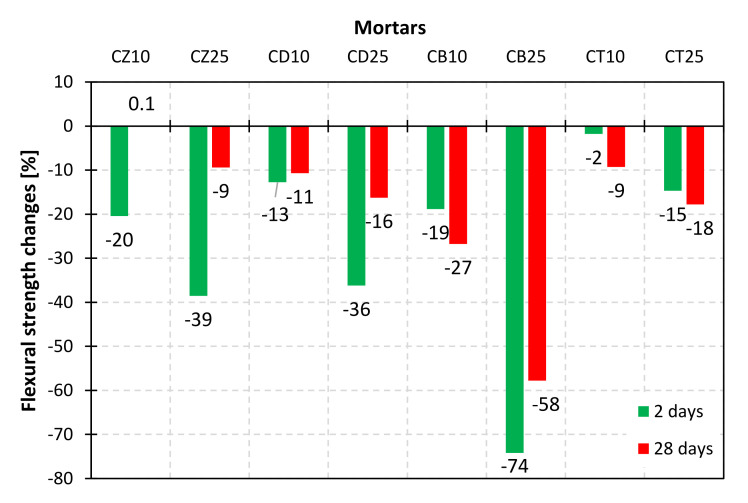
Relative changes in flexural strength after 2 and 28 days of hydration, in comparison to the reference mortar (Cref).

To investigate the impact of mineral additives on the mechanical performance of composites obtained with the use of modified cements, mortar bars were subjected to early (2 days) and standard (28 days) compressive and flexural strength examinations. The results are presented in Figure 12, Figure 13, Figure 14 and Figure 15.

Research showed that bentonite was the least favorable among all tested mineral additives. When used as a substitute for 10% and 25% of OPC in the mortar, it was responsible for a 30% and 80% drop in compressive strength, respectively. The obtained values were much lower than those reported by Ahmad et al. [26], as the material was not thermally treated. The results varied depending on the hydration time. After two days of curing, a deterioration of early compressive strength was observed for all studied materials. For the 10% addition of diatomite and trass, the decrease was insignificant (below 2%), which indicates their high chemical activity. After 28 days of curing, the strength of the mortar containing 10% of trass in the binder was comparable to the reference sample, while for the same addition of diatomite, it was 11% lower. This outcome stands in opposition to the results obtained by Degirmenci et al. [30], who observed a 52% drop in the strength of mortars after 28 days for the same diatomite content. The difference in the obtained values leads to the conclusion that diatomites of various origin are characterized by different pozzolanic activity and specific surface area. In the case of zeolite, the beneficial effect of the pozzolanic reaction is clearly visible–after 28 days, despite the earlier decrease, a 7% increase in strength was observed for the CZ10 sample, which corresponds to the results obtained by Ahmadi et al. [23] and Ghafari et al. [24]. The strength of the mortar with the 25% cement substitution matched the reference sample. The obtained strength values correlate with the thermal analysis results-for zeolite, the Ca(OH)_2_ content was the lowest and the chemically bound water content was the highest, indicating an ongoing pozzolanic reaction. For all the materials studied, the correlation between mechanical strength and chemically bound water content is, however, nonlinear. This can be explained by the fact that water can be partially adsorbed or absorbed by the mineral additives. Another reason could be the difference in activity resulting from the varying specific surface area. Moreover, it is possible that the products of the pozzolanic reaction of each mineral additive are different and characterized by various molar ratios of water in the structure, e.g., due to the changing CaO/SiO_2_ and Al_2_O_3_/SiO_2_ molar ratios, which has already been reported in the literature [35].

The results of flexural strength evaluations allow us to draw similar conclusions. From the point of view of beneficial influence on the mechanical properties of composites, the mineral additives could be ranked as follows: bentonite < diatomite, zeolite < trass after 2 days and bentonite < diatomite < trass < zeolite after 28 days of curing.

## 4. Conclusions

The article presents a comparison of alternative mineral additives, relatively rarely used in the cement and concrete industry. The conclusions drawn from the research are of great importance, as the potential wide scale utilization of these materials would allow to produce more sustainable binders and reduce the carbon footprint related to building materials manufacturing. Therefore, comparing a variety of materials under the same experimental conditions is valuable not only from the scientific, but also from the practical point of view, as such an approach makes it easier for companies to utilize the results on an industrial scale.

The following conclusions were drawn from the research:

- The most water-demanding mineral additive was bentonite, while trass, zeolite and diatomite increased the water demand by only 2–4 percentage points for 10% and 7–11 percentage points for 25% of the addition.
- The initial setting time of cements modified with trass and zeolite was 5–30 min shorter, compared to the reference cement. Diatomite increased the setting time by 35 min for 10% and 65 min for 25% of the addition. The longest setting time was recorded for the bentonite-containing binder. All cements met the requirements of the EN 197-1 standard.
- All of the studied mineral additives decrease the consistency of the fresh mix, which exhibited in the reduced flow of standard mortars both 5 and 40 min after mixing. Mortar modified with bentonite was the driest. Zeolite, trass and diatomite reduced the flow in the range of 12–22% for 10% and 16–24% for 25% of the addition, respectively.
- Alternative mineral additives change the kinetics of heat evolution, as evidenced by the additional exothermic effect (aluminate effect) observed in the calorimetric curves. Nevertheless, for the 10% addition of the SCMs, no significant changes were observed in the total heat evolved after 41 h of hydration. In the case of samples containing 25% of zeolite and diatomite, there was an approx. 10% increase in the cumulative heat of hydration, compared to reference cement with the same addition of inert material (ground quartz sand).
- Thermal analysis of the pastes showed that after 28 days of curing, modified cements yield a higher amount of hydration products, compared to the reference samples; however, no linear correlation was found between the content of chemically bound water and the mechanical strength of the mortars. The reason should be sought in the surface area characteristics and the possible water absorptivity of the materials, although further research is required in order to find a full explanation.
- Early compressive strength of mortars modified with alternative mineral additives was lower, compared to the reference sample. After 28 days of curing, a significant increase in strength was observed in the case of zeolite-containing mortar. Diatomite and trass have also considerably improved the late mechanical strength of the composites, which proves the pozzolanic character of these materials.

## Figures and Tables

**Table 1:** Selected ASTM C618 requirements for natural pozzolans (Class N).

Chemical requirements	
SiO_2_ + Al_2_O_3_ + Fe_2_O_3_, min. %	70.0
SO_3_, max. %	4.0
Moisture content, max. %	3.0
Loss on ignition, max. %	10.0
**Physical requirements**	
Fineness. Amount retained when wet-sieved on 45 µm (No. 325) sieve, max. %	34.0
Strength activity index with Portland cement at 7/28 days, min. % of control	75.0/75.0 (Meeting either indicates specification compliance)
Water requirement, max. % of control	115.0
Soundness. Autoclave expansion or contraction, max. %	0.8

**Table 2:** Mix proportions of pastes.

Sample	Pastes						
	Cement [g]	Mineral Additive [g]				Ground Sand [g]	Water [g]
	CEM I 42.5R	Z	D	B	T		
Cref	5.00	-					2.50
Cref10	4.50	-				0.50	2.50
Cref25	3.75	-				1.25	2.50
CZ10	4.50	0.50	-	-	-	-	2.50
CZ25	3.75	1.25	-	-	-		2.50
CD10	4.50	-	0.50	-	-		2.50
CD25	3.75	-	1.25	-	-		2.50
CB10	4.50	-	-	0.50	-		2.50
CB25	3.75	-	-	1.25	-		2.50
CT10	4.50	-	-	-	0.50		2.50
CT25	3.75	-	-	-	1.25		2.50

Ref—reference (OPC), Z—zeolite, D—diatomite, B—bentonite, T—trass.

**Table 3:** Mix proportions of mortars.

Sample	Mortars							
	Cement [g]	Mineral Additive [g]				Water [g]	Sand [g]	SP [%]
	CEM I 42.5R	Z	D	B	T			
Cref	450	-				225	1350	0.0
CZ10	405	45	-	-	-	225	1350	0.0
CZ25	338	112	-	-	-	225	1350	0.0
CD10	405	-	45	-	-	225	1350	0.0
CD25	338	-	112	-	-	225	1350	2.0
CB10	405	-	-	45	-	225	1350	6.0 *
CB25	338	-	-	112	-	225	1350	20.0 *
CT10	405	-	-	-	45	225	1350	0.0
CT25	338	-	-	-	112	225	1350	0.0

* exceeding the dosage recommended by the manufacturer. Ref—reference (OPC), Z—zeolite, D—diatomite, B—bentonite, T—trass.

**Table 4:** Standard properties of CEM I 42.5R.

Property	Value
Water demand [%]	28
Initial setting time [min]	160
Density [g/cm^3^]	3.12
Blaine’s specific surface area [cm^2^/g]	3500

**Table 5:** Chemical composition of cement and mineral additives.

Oxide:	CEM I 42.5R	Zeolite (Z)	Diatomite (D)	Bentonite (B)	Trass (T)
CaO	68.38	3.53	0.49	2.60	3.93
MgO	0.81	0.80	1.08	3.38	1.30
SiO_2_	18.00	76.28	76.69	66.57	56.80
Al_2_O_3_	4.46	12.81	12.98	19.14	20.39
Na_2_O	0.27	0.70	0.52	4.15	3.95
K_2_O	0.58	3.43	1.92	0.80	5.82
Fe_2_O_3_	2.61	1.83	4.75	2.68	5.88
SO_3_	4.19	0.05	0.66	0.16	0.21
Cl	0.05	0.07	0.02	0.03	0.06
P_2_O_5_	0.11	0.05	0.12	0.03	0.22
Total	99.45	99.53	99.21	99.54	98.55

**Table 6:** Physical properties of mineral additives.

Material	Zeolite (Z)	Diatomite (D)	Bentonite (B)	Trass (T)
Density [g/cm^3^]	2.29	2.31	1.05	2.52
Humidity [%]	2.47	3.04	6.17	1.46
BET specific surface area [m^2^/g]	31.49	23.64	38.65	22.33

## Data Availability

The data presented in this study are available on request from the corresponding author.
